# Giant cell arteritis: A case report and review of literature

**DOI:** 10.1016/j.radcr.2021.08.072

**Published:** 2021-10-02

**Authors:** Matthew A. Crain, Dhairya A. Lakhani, Lana Winkler, Ayodele Adelanwa, Cathy Kim

**Affiliations:** a West Virginia University School of Medicine, Morgantown, WV; b Department of Radiology, West Virginia University, 1 Medical Center Drive, Morgantown, WV, 26506; c Department of Pathology, Ruby Memorial Hospital, West Virginia University, Morgantown, WV

**Keywords:** Giant cell arteritis, Vasculitis, Inflammatory vasculitis, GCA, Giant Cell Arteritis, Coronary Artery Bypass Graft, (CABG), CT, Computerized Tomography

## Abstract

Giant cell arteritis, the most common form of vasculitis in the elderly, is characterized by granulomatous inflammation of arteries, which can lead to serious, life-threatening conditions including aortic aneurysms, ruptures, and dissections as well as blindness. Since GCA can be treated by immunosuppressant therapy, such as corticosteroids, early diagnosis and treatment may reduce the risk of serious disability and morbidity. While temporal artery biopsy is considered the gold standard to diagnosis giant cell arteritis, it is intrusive with inherent risks as well as unreliable due to tissue sampling. Imaging studies, such as computerized tomography, are nonintrusive and have been shown to identify vasculitis including giant cell arteritis. We present a case of a 72-year-old male patient who was diagnosed with giant cell arteritis by temporal artery biopsy during surgery for aortic aneurysm and coronary artery bypass graft. Computerized tomography imaging studies, prior to the surgery and biopsy, were suggestive of vasculitis. This case serves to emphasize the beneficial role of imaging studies to assess vasculitis, including giant cell arteritis, that can be done prior to the progressive development of more serious debilitating and potentially fatal pathology.

## Background

Giant cell arteritis (GCA) is a chronic vasculitis, characterized by granulomatous inflammation of arteries, which can lead to serious, life-threatening conditions including aortic aneurysms, ruptures, and dissections as well as blindness [1], [2], [3], [4], [5]. Clinical symptoms of GCA are often nonspecific, such as headaches, chest pain, fatigue, visual disturbance, and weight loss [1]. Since GCA affects older adults over 50 years with a peak incidence between 70 and 80 years, these symptoms are common among the elderly and could reflect other conditions. Therefore, GCA is frequently misdiagnosed, even though it is the most common form of vasculitis in this population [1].

Fortunately, immunosuppressant therapy, such as corticosteroids, is often effective in reducing the inflammation associated with GCA and, therefore, its progression and the risk of serious and, at times, life-threatening conditions as well as the need for surgical interventions [6]. Therefore, it is critical to diagnose and treat GCA as early as possible. While temporal artery biopsy (TAB) is considered the diagnostic gold standard for GCA, it is intrusive and often done when the disease process has already seriously progressed [1]. In addition, since TAB involves sampling of arterial tissue, it is not always reliable [7]. Imaging studies provide a less intrusive, and potentially more reliable method of detecting signs of GCA and other forms of vasculitis, which could lead to early treatment with corticosteroids [7], [8], [9], [10]. This course of early diagnosis and treatment, if effective, might not only confirm the role of aortic inflammation and diagnosis of GCA, but also prevent the more serious, potentially disabling and fatal consequences of untreated GCA.

We present a case in which GCA was diagnosed by TAB during surgery for aortic aneurysm and coronary artery bypass graft (CABG) in a 72-year-old male patient. CT imaging studies, prior to the surgery and biopsy, were suggestive of vasculitis. This case serves to emphasize the beneficial role of imaging studies to assess GCA that can be done prior to the progressive development of more serious debilitating and potentially fatal pathology.

## Case report

**Fig. 1:**
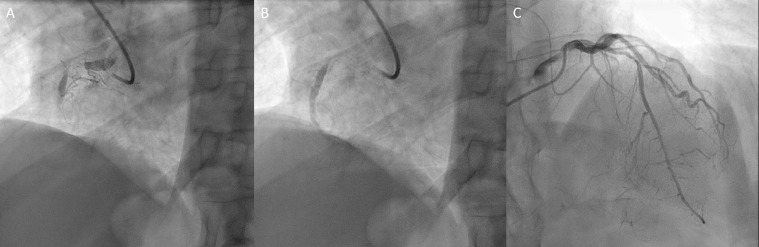
Coronary angiogram showed advanced coronary artery disease of right coronary artery (A and B) and left anterior descending artery (C)

A 72-year-old man with essential hypertension, hyperlipidemia, and type II diabetes mellitus was referred for coronary artery bypass graft (CABG) evaluation. He presented at outside facility with an acute exacerbation of chronic chest pain, exertion dyspnea, and fatigue. Coronary workup at outside facility included chest radiograph, computed tomography (CT) chest with contrast, transthoracic echocardiogram (TTE) and coronary angiogram (CAG). CT chest was reported as no acute abnormalities at outside facility. CAG showed advanced coronary artery disease of left anterior descending and right coronary artery (Fig. 1). Patient was subsequently referred to our facility for CABG evaluation.

**Fig. 2:**
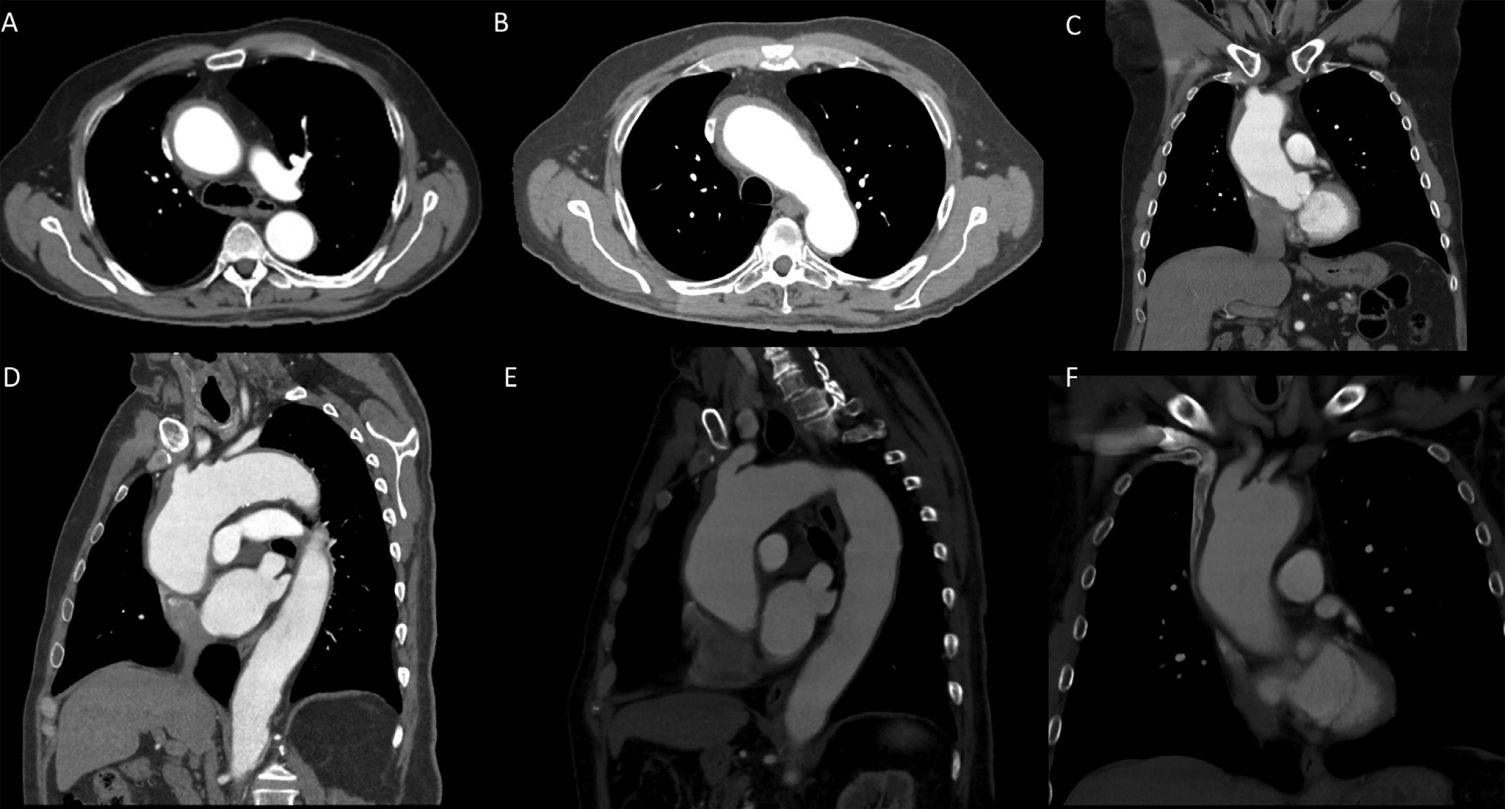
Review of outside CT chest images revealed ascending thoracic aortic aneurysm (5.3 cm in largest diameter). In addition, circumferential soft tissue attenuation thickening was noted involving the aortic root, predominately in the ascending thoracic aorta and the proximal aortic arch. A small ulceration in the aortic arch between the brachiocephalic artery and left common carotid artery was also noted. The arch vessels were normal. Constellation of these findings without significant atherosclerotic vascular disease and intimal calcification were suggestive of aortic vasculitis, specifically giant cell arteritis (GCA)

At our institute, on arrival he was afebrile and had stable vitals. Initial pre-operative workup was unremarkable. Review of outside CT chest images revealed ascending thoracic aortic aneurysm (5.3 cm in largest diameter). In addition, circumferential soft tissue attenuation thickening was noted involving the aortic root, predominately in the ascending thoracic aorta and the proximal aortic arch. A small ulceration in the aortic arch between the brachiocephalic artery and left common carotid artery was also found. The arch vessels were normal (Fig. 2). These findings, together with lack of atherosclerotic disease and intimal calcification, were suggestive of aortic vasculitis, specifically giant cell arteritis (GCA).

**Fig. 3:**
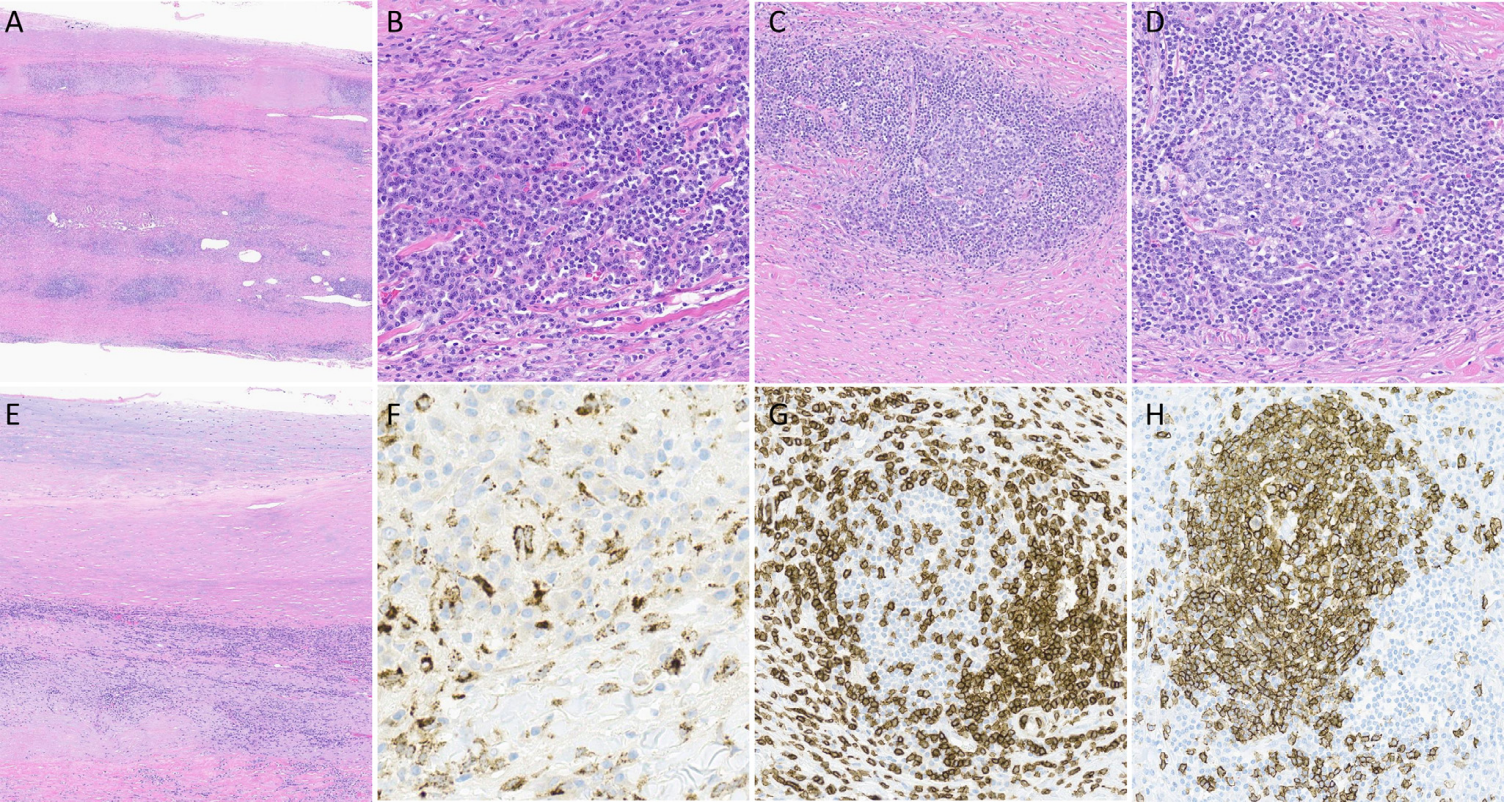
A: Section of aorta demonstrating significant inflammation composed of lymphocytes, histiocytes, occasional giant cells and several lymphoid follicles. H&E (10x). B: Admixture of lymphocytes and plasma cells within the wall of the aorta. H&E (200x). C: One of many lymphoid follicles within the wall of the aorta demonstrating a distinct germinal center. H&E (100x). D: One of many lymphoid follicles within the wall of the aorta demonstrating a distinct germinal center. H&E (200x). E: Section of aorta demonstrating transmural inflammation composed of lymphocytes, histiocytes, occasional giant cells and several lymphoid follicles. H&E (50x). F: CD68 immunohistochemical stain highlights the histiocytes. (400x). G: CD3 immunohistochemical stain highlights T lymphocytes in the lymphoid follicle. (200x). H: CD20 immunohistochemical stain highlights the B lymphocytes in the lymphoid follicle. (200x)

Patient was subsequently taken to the OR for CABG and during the surgery, biopsies were taken from the aorta, which showed a marked inflammatory infiltrate with numerous lymphocytes, lymphoid follicles with germinal centers, histiocytes, and occasional giant cells (Fig. 3). These pathology findings confirmed the diagnosis of vasculitis, specifically, giant cell arteritis (GCA).

The patient recovered well from the surgery and was discharged the following week, with outpatient follow-up.

## Discussion

Giant cell arteritis (GCA) is the most common form of vasculitis in the elderly with presenting symptoms that are common and nonspecific including headaches, bodily pains, fatigue, and visual disturbance. However, untreated GCA is known to increase risk for the development of more serious conditions, including aortic aneurysms, ruptures, and dissections that can lead to serious disability and sudden death as well as blindness. Therefore, without early identification and diagnosis, GCA can be the underlying cause of serious progressive cardiothoracic disease in the elderly. When diagnosed early, GCA can often be effectively treated with immunosuppressant therapy, including corticosteroids, thereby reducing the risk of progressive cardiothoracic disease.

The gold standard for diagnosing GCA is temporal arterial biopsy (TAB). However, this procedure is intrusive and typically performed during surgery, once the cardiothoracic disease progressed to the point of requiring surgical intervention, as in the case of our patient. TAB has inherent risks of complications and may lead to false negatives given the nature of biopsy sampling. Preferably, diagnosis of GCA would be made earlier, more reliably, and with less intrusive procedures, leading to a trial of immunosuppressant therapy prior to the need for surgical intervention. The identification of possible vasculitis in the pre-op review of our patient's CT images demonstrates the potential clinical benefit of radiologic imaging studies to assess for GCA.

Duffner *et al.* performed a systematic literature review, through mid-2017, of imaging techniques for the diagnosis, outcome prediction, and disease monitoring in large vessel vasculitis [11]. They summarized 39 GCA studies, which found support for the use of US and MRI of temporal arteries with pooled sensitivity of 73 and 77% sensitivity, respectively, as well as pooled specificities of 96% and 88%, compared with clinical or TAB diagnosis. More limited studies of CT and CTA were reviewed by them, though at least one revealed CT- angiography (CTA) sensitivity of 73% and specificity of 78%.

In a 2017 analysis of CT imaging studies, comparing 64 GCA patients with 43 polymyalgia rheumatica patients and 67 controls, Berthod *et al.* found that the aortic wall thickness was significantly greater in GCA patients [12]. Using a receiver operating characteristic curve, they showed that a wall thickness of 2.2 mm was the optimal threshold to diagnose GCA. Aortic diameters and atheroma scores were similar among the groups. Their findings are consistent with the aortic wall thickness of our patient with GCA.

In a 2018 review of imaging studies for the diagnosis of GCA, Halbach *et al.* did a systematic comparison of the benefits and weaknesses comparing TAB with ultrasound (US), magnetic resonance imaging (MRI), and positron emission tomography (PET) scanning [7]. They argued that arterial US of patients with a high pretest probability for GCA can be used with nearly 80% sensitivity to confirm the diagnosis or guide the best location for biopsies, based on characteristic vascular halo as well as degree of stenosis and occlusion. Since US is a widely used, low-cost, and nonintrusive imaging modality, they recommended its use for elderly patients with presenting symptoms consistent with GCA. MRI can also be used to identify the presence of arterial inflammation and wall thickening as well as vessel stenosis, occlusions, dilations, and aneurysms of large vessels throughout the region more systematically than TAB sampling. However, MRI is expensive and has not been formally studied to diagnose GCA. Halbach *et al.* also make a case for FDG-PET and PET/CT for the diagnosis of GCA, though it is also expensive and may not be as reliable as other imaging modalities.

Similarly, Berger *et al.* emphasized the clinical benefits of imaging in the diagnosis and treatment of GCA [8]. Their 2018 review of the literature concluded that color duplex sonography (CDS) is particularly sensitive to assess the vascular wall anatomy as well as the lumen and blood flow. They also compared the relative benefits and weaknesses of CT, CTA, MRI, and PET in the diagnosis of vasculitis including GCA. Since immunosuppressants can rapidly reduce vascular inflammation and, therefore, detection of vasculitis, they suggested that imaging studies should be done prior to initiating treatment. Berger *et al.* argued that, since invasive TAB is diagnostic in only 50% of patients, "imaging is essential for diagnosing GCA." They recommended the development of "fast-track clinics with diagnostic algorithms adopted to local expertise" for imaging studies that are readily available [8].

Schafer *et al.* conducted an extensive literature review from 2015 - 2020 of imaging studies for the diagnosis, monitoring, and outcome prediction of large vessel vasculitis including GCA [9]. They concluded that "imaging has partly replaced histology for confirming large vessel vasculitis" since it is nonintrusive with minimal risk and has "excellent diagnostic accuracy" provided that the radiologist has adequate training and experience. Studies using US, MRI, and CT have found clear and consistent signs of vasculitis manifested by a homogeneous arterial wall thickening, which is mostly concentric, as seen in the CT imaging of the presenting patient. The authors recommended US as the first imaging modality since there is no radiation, followed by imaging with CT, MRI, and PET/CT. Given the complexity of integrating and interpreting imaging analyses, together with history and clinical examination, they indicated that further research is needed to evaluate the role of imaging and outcome measurement as well as improved algorithms and quantitative analyses.

In a 2021 retrospective study of GCA patients diagnosed over a 20-year period who had vascular imaging evaluation when diagnosed, de Mornac *et al.* analyzed the risk factors for symptomatic vascular events [13]. Among other clinical factors, abnormal CT imaging of the thoracic aorta was an independent predictor of subsequent serious complications including strokes, ischemia, aortic surgery, and death. Their findings underscore the importance of early large-vessel imaging assessment for elderly patients presenting with clinical symptoms that could reflect GCA. This research suggests that earlier detection of GCA using CT and other imaging modalities might have reduced the likelihood of the serious vascular events experienced by our patient.

GCA requires an early diagnosis to avoid serious disability and morbidity in the elderly population. A missed diagnosis can have devasting effects. While TAB continues to be the gold standard for the diagnosis of GCA, it requires an invasive procedure with associated risks as well as inconsistent reliability due to biopsy sampling. The serious pathology seen in our patient, reflecting a progressive disease process likely due to underlying GCA, might have been reduced if the diagnosis could have been made earlier and immunosuppressant treatment initiated. Early assessment with noninvasive imaging scanning is recommended in elderly patients who present with clinical symptoms associated with GCA. Further research is needed in order to determine the best imaging modalities and algorithms to effectively diagnosis GCA and other forms of vasculitis.
